# Extracellular vesicles in Graves’ disease and Graves’ orbitopathy: immunoregulatory mechanisms, biomarkers, and therapeutic potentials

**DOI:** 10.3389/fimmu.2026.1762376

**Published:** 2026-01-30

**Authors:** Yuqing Chen, Ying Yang, Shuo Yang, Wenjie Yuan, Lihong Jiang, Ruili Wei

**Affiliations:** 1 Department of Ophthalmology, Changzheng Hospital of Naval Medical University, Shanghai, China; 2 Department of Ophthalmology, Wuxi No. 2 People’s Hospital, Wuxi, Jiangsu, China; 3 Shanghai Jingye High School, Shanghai, China; 4 Department of Ophthalmology, Shanghai General Hospital, Shanghai Jiao Tong University, Shanghai, China; 5 National Clinical Research Center for Eye Diseases, Shanghai, China; 6 Shanghai Clinical Research Center for Eye Diseases, Shanghai Key Clinical Specialty, Shanghai, China

**Keywords:** exosomes, extracellular vesicles, Graves’ disease, Graves’ orbitopathy, immunomodulation

## Abstract

Graves’ disease (GD) is a common autoimmune thyroid disorder and is often accompanied by Graves’ orbitopathy (GO), an inflammatory eye disease that can significantly reduce the quality of patients’ life. Despite understanding of GD and GO has progressed, the mechanisms driving disease progression remain incompletely defined. Emerging evidence highlights extracellular vesicles (EVs), particularly exosomes, as important mediators of immune regulation and tissue remodeling in autoimmune disorders, including GD and GO. This review summarizes current knowledge of EVs biogenesis and molecular compositions, highlighting their contributions to GD and GO pathogenesis. We also discuss the diagnostic and prognostic potential of EV-associated miRNAs and proteins, and consider findings from other immune-mediated ocular diseases to place these observations in a broader immunopathological context. Overall, EVs appear to be actively involved in GD and GO and may serve as useful tools for disease monitoring and therapy development. Nonetheless, challenges such as methodological variability and limited functional validation remains. Standardized protocols and larger, multicenter studies are needed to support the clinical translation of EV-based approaches.

## 1 Introduction

Graves’ disease (GD) is an autoimmune disorder characterized by hyperthyroidism, diffuse goiter, and autoantibodies against the thyroid-stimulating hormone receptor (TSHR) (1). It is the most common cause of hyperthyroidism in the world. Epidemiological studies indicate a lifetime prevalence of approximately 0.5% and 3% in men and women, respectively, which demonstrates significant gender susceptibility differences (2). Graves’ orbitopathy (GO), also referred to as thyroid eye disease, is an extrathyroidal manifestation of GD (3–5). GO affects around 25-30% of people with GD, while advanced orbital imaging techniques can detect about 70% of cases (6–8). GO presents with inflammation of the extraocular muscles, orbital fat expansion and posterior tenon’s capsule involvement leading to proptosis, diplopia, eye pain, optic neuropathy and blindness (9, 10).

Current treatments for GO mainly target inhibiting immune activity during the active disease period. Current first-line treatment with glucocorticoid pulse therapy for patients with moderate-to-severe active GO shows limited efficacy against fibrotic changes in the orbital tissues (11). Meanwhile, the monoclonal antibody Teprotumumab, which targets IGF-1R, is highly expensive and has limited accessibility (12). Despite historic advances in current treatments for GD, the underlying pathogenesis remains incompletely understood. GD and GO share common immune pathways, primarily involving TSHR and IGF-1R autoantibodies, T-cell activation, and proinflammatory cytokine-mediated proliferation of orbital fibroblasts(OFs). Early diagnosis and timely interventions are important for improving treatment outcomes (13). At present, the clinical diagnosis of GD is largely based on the Bartley criteria, which integrate ocular signs with thyroid function assessment (14). However, serological tests and imaging are not suitable for long-term monitoring, underscoring the need for more reliable and stable biomarkers. Recent studies have highlighted extracellular vesicles (EVs) as important mediators in immune regulation and intercellular communication (15, 16). EVs can carry bioactive substances including nucleic acids, proteins, lipids, participating in the regulation of immune processes and cellular behaviors (17). Given their heterogeneous size, cellular origin, and molecular contents, EVs show considerable capacity to modulate cellular phenotypes and immune responses. In autoimmune diseases such as GD (18) and GO (19), EVs may convey pathogenic signals, making them attractive candidates as biomarkers and potential therapeutic targets. This review summarizes existing evidence and provides recommendations for future research in these emerging fields.

## 2 Extracellular vesicles

EVs belong to a group of diverse lipid bilayer-enclosed particles that are secreted by a variety of cells. EVs do not have autonomous replication ability, but act as intercellular messengers for the transport of bioactive molecules such as nucleic acids, proteins, lipids, and metabolites. Based on their biogenesis pathways and particle size, EVs can be classified into three main categories: exosomes (30–150 nm), microvesicles (100-1,000 nm), and apoptotic bodies (500-2,000 nm), respectively (20–22).

### 2.1 Biogenesis and characteristics

**Figure 1:**
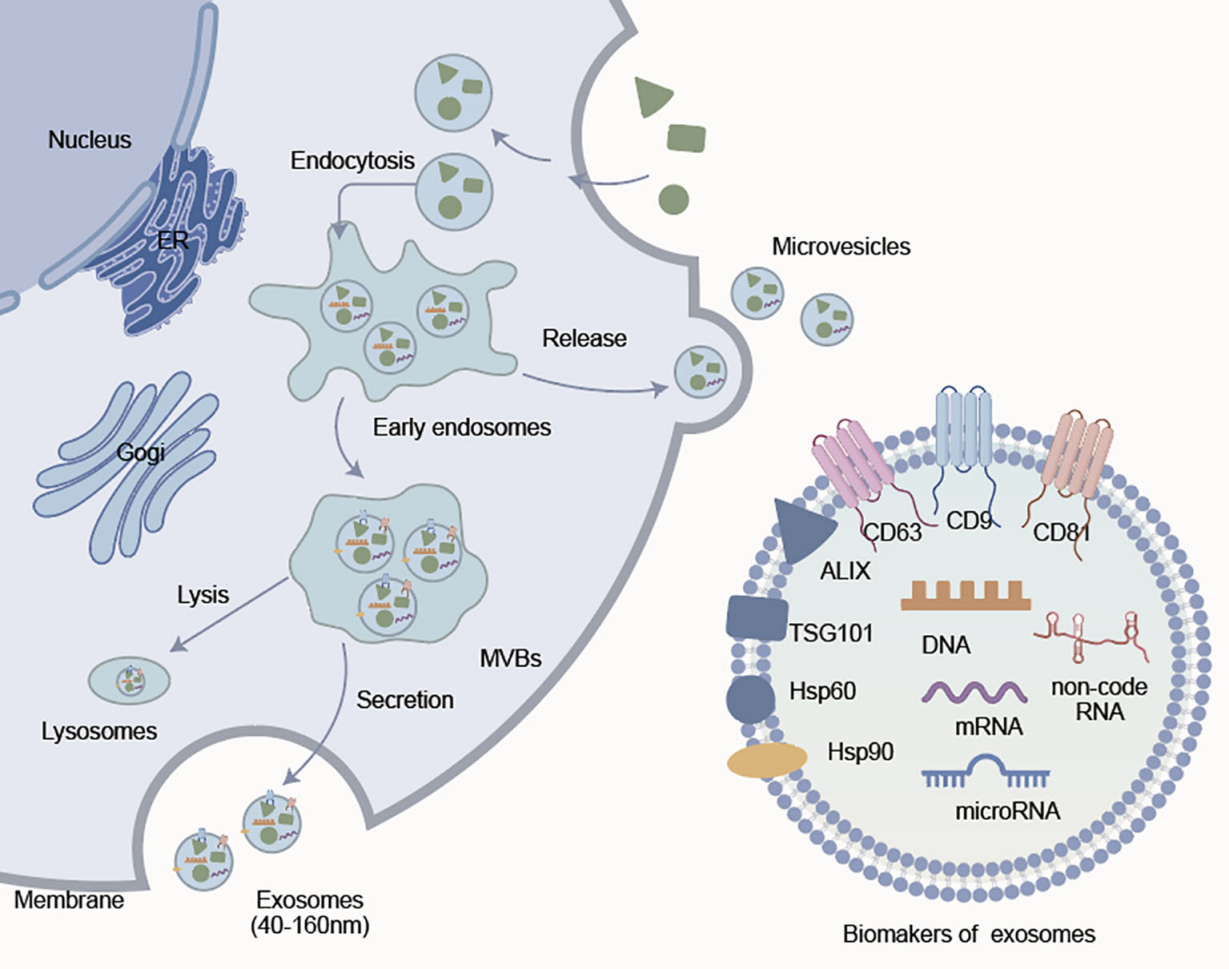
Formation and molecular features of extracellular vesicles. Exosomes (40–160 nm) are generated through the endosomal pathway, involving early endosomes, MVBs, and secretion through membrane fusion. The microvesicles are formed through direct budding from plasma membrane. Both types carry functional molecules, such as DNA, mRNA, non-coding RNA, and proteins. Common exosome markers include CD9, TSG101, CD81, CD63, ALIX, Hsp60, and Hsp90, which help define their identity and biological functions.

EVs are grouped by biogenesis route. Exosomes (40–160 nm) are endosomal-derived. are formed by the inward budding of the plasma membrane to produce early endosomes and MVBs. As MVBs fuse with the plasma membrane, ILVs are released from MVBs as fully developed exosomes. Conversely, microvesicles (50–1000 nm) or ectosomes, are produced by direct outward budding of the plasma membrane (**Figure 1**) (20, 23). Exosomes and microvesicles may have some overlap in their size range but have different origins, structures, and surface markers (24–26). Exosomes express tetraspanins (CD9, CD63, CD81), ALIX, TSG101, and heat shock proteins (Hsp60, Hsp90) as established identifiers (27, 28). Annexin A1 is emerging as a candidate distinguishing marker for microvesicles (29). Nevertheless, a universal classification system of EVs has not yet been established and thus more studies are required to identify definitive subtype-specific markers.

### 2.2 Composition and functions

EVs play key roles in the transfer of cargo between cells, either as direct delivery vehicles for functional cargo that is transferred to a recipient cell or via activation of surface receptors on recipient cells that initiate intracellular signaling (30, 31). The transported cargo is proteins (surface/cytosolic), DNA, mRNA, microRNAs, amino acids, and metabolites (32, 33). These molecules take part in some different processes like immune system adjustment, inflammatory reaction, angiogenesis, and tissue repairments (34–38). Among these exosomal components, exosomal microRNAs are of particular significance as regulators and possible biomarkers for autoimmune and inflammatory diseases (35, 39, 40). EVs show advantageous properties for biomedical applications, such as structural stability, low immunogenicity, and high biocompatibility. These characteristics support their role as a next generation vector for target drugs delivery (41, 42). Recently, studies employing more refined purification methods have called into question earlier findings about the presence of double-stranded DNA and histones in EVs. The current evidence suggests that canonical small EVs do not contain dsDNA or DNA-bound histones, which contradicts earlier assumptions (42, 43). And this also highlights the critical importance of clearly defining the specific composition of EVs in both scientific studies and clinical applications.

### 2.3 Immunomodulatory properties

EVs that are released by immune cells are related to antigen presentation, T cell activation, and regulation of immune tolerance (44, 45). In addition to immune cells, EVs from nonimmune cells are also involved in immunity. Tumor-derived EVs can induce apoptosis of immune cells and inhibit T cell activation, which helps tumor cells to evade immune attack (46). Inflammatory conditions improve the immunosuppressive properties of mesenchymal stem cell-derived EVs, which control inflammation by favoring Treg differentiation and altering macrophage polarization (47–49). These observations highlight the context-dependent immunoregulatory nature of EVs, which are derived from cells and are surrounded by a microenvironment. A specific EVs’ role in immune mediated eye disease has yet to be investigated. Future research should aim for mechanistic studies with standardized protocols to investigate their potential for treatment and diagnostics.

### 2.4 Isolation of exosome

Exosomes are nano-sized extracellular vesicles distributed through vastly complex body fluids, which makes high-yield exosome isolation challenging (50). Although ultracentrifugation is considered the “gold standard” for exosome separation owing to its high processing capacity, it has notable limitations. The primary challenge is the high content of protein aggregates and lipoproteins present in the sample. Since this method cannot completely separate exosomes from other components, these impurities significantly compromise the accurate quantification of exosomes and subsequent analyses (51). These limitations have driven the development of diverse separation techniques to meet higher exosome purification demands, including traditional methods such as ultrafiltration, size exclusion chromatography, polymer precipitation, and immunoaffinity approaches, alongside emerging methods like microfluidics, DNA aptamer affinity, fluid flow-based separation, thermophoresis, and lipid recognition separation, each with its own unique advantages and disadvantages (52).

## 3 EVs in Graves’ orbitopathy

It is found that there is significant evidence for the role of EVs in the development of GO, especially exosomes. These vesicles which isolated from blood and tears of GO patients, contain many different bioactive molecules that can regulate intraocular inflammation, fibrosis, and serving as biomarkers for therapeutic response to drug treatments.(**Table 1**).

**Table 1:** Summarization of studies investigating the role of EVs in GO pathogenesis.

Origin of EVs	Cargo of EVs	Biological function	EVs isolation method	Group and sample size	Year	Reference
GO patients’ serum-derived Exos	IGF-1R and HSP60	Interacted with PBMCs via TLR2/3 and activated the MyD88/TRIF/NF-κB pathway, upregulating IL-6 and IL-1β, suggesting a pro-inflammatory mechanism.	differential ultracentrifugation	GO patients (n=7), GD patients (n=26), HC (n=26)	2021	(53)
GO patients’ tear-derived Exos	IL-6`IL-8`MCP-1	Promoted inflammation and tissue remodeling.	Polymer Precipitation	GO patients (n=8), HC (n=8)	2021	(54)
Patients’ (improve following ivGC therapy) plasma Exos	miR-885-3p	Enhanced glucocorticoid sensitivity in OFs through blocking AKT/NF-κB signaling pathway.	Polymer Precipitation	Significant improvement patients (n=11), non-significant improvement patients (n=6)	2022	(55)
GO patients’ tear-derived Exos	Caspase-3, complement C4A, and apolipoprotein A-IV	Mirrored expression patterns in orbital tissues.	membrane-based spin column	GO patients (n=24), GD patients (n=24), HC (n=16)	2022	(56)
GO patients’ serum and tear-derived Exos	IL-1 and IL-18	Participated in immune response, apoptosis, complement activation, and lipid metabolism	membrane-based spin column	GO patients (n=24), GD patients (n=24), HC (n=16)	2022	(56)
Active GO found-patients’ plasma Exos derived Thy-1+ orbital fibroblasts	Unmentioned	Induced higher levels of IL-1β, IL-6, and hyaluronic acid.	Size Exclusion Chromatography	Active GO patients (n=25), HC (n=25)	2024	(57)
Active GO found-patients’ plasma Exos and peripheral blood mononuclear cells	miR-144-3p	Intensified inflammatory and fibrotic responses in OFs and further inhibited proliferation.	Size Exclusion Chromatography	Active GO patients (n=25), HC (n=25)	2024	(57)

### 3.1 EVs as immune activators in GO

A mechanism is the immunostimulatory activity of EVs in GO. Serum-derived exosomes from the GO and GD display higher levels of insulin-like growth factor-1(IGF-1) receptor and heat shock protein 60 (HSP60), these components relate to autoimmune reactions. Due to the lipid bilayer structure of exosomes, GD-EXO can directly fuse with peripheral blood mononuclear cells (PBMCs) or be uptaken by PBMCs through binding to Toll-like receptors (TLRs) on the cell surface via the HSP60 protein it carries. Research has shown that GD-EXO can be taken up by PBMCs from healthy donors and undergo binding with PBMCs, while increasing the proportion of CD11c^+^TLR2^+^ and CD11c^+^TLR3^+^ dendritic cells. Furthermore, antigens and pathogenic factors within the exosomes are transported into PBMCs, activating MyD88 and TRIF, leading to enhanced NF-κB p65 phosphorylation. This ultimately promotes the release of IL-6 and IL-1β, inducing inflammatory responses and contributing to the pathogennesis of GD. However, due to the relatively small number of patients with early-onset GO outpatient who were not receiving treatment, this study regrettably could not proceed with the subsequent GO-EXO functional research. Subsequently, subject to patient availability and experimental conditions permitting, more in-depth research on the role of GO patients’ exosomes in immune and inflammatory responses need to be conducted in future research (53).

### 3.2 miRNA cargo and fibrotic signaling

Exosomal miRNAs, particularly miR-144-3p, enhance fibrotic and inflammatory responses in OFs, suggesting shared pathological pathways in fibrosis induction across different etiologies. Li et al. demonstrated that miR-144-3p was significantly upregulated in both Pla-Exos and PBMCs during the active phase of GO. Subsequently, miR-144-3p mimics and control mimics were transfected into Thy-1+ OFs, followed by assessment of relevant target mRNAs (inflammatory molecules, HAS1, HAS2, HA) and cell proliferation. Results demonstrated that Pla-Exos derived from active-phase GO patients activated Thy-1+ fibroblasts, inducing expression of inflammatory cytokines and pro-fibrotic markers. Furthermore, these exosomes inhibited proliferation of Thy-1+ OFs. Compared to normal human Pla-Exos, active-phase GO Pla-Exos significantly upregulated the expression and secretion of pro-inflammatory factors (e.g., IL-1β, IL-6, TNF-α, CXCL2, and RANTES) in Thy-1+ OFs. As key mediators of inflammation and fibrosis, the upregulation of these cytokines and chemokines promotes the recruitment of immune cells to orbital connective tissue and enhances hyaluronic acid accumulation in the extracellular matrix, thereby perpetuating inflammation and fibrosis in GO (57).

### 3.3 EVs as predictive biomarkers for therapeutic response

Given the variable responsiveness of GO patients to intravenous glucocorticoid (ivGC) therapy, studies have explored exosomes as biomarkers to predict the treatment outcomes. Analysis revealed that miR-885-3p as a potential biomarker indicating therapeutic response. In GO patients who showed significant symptom improvement after IVGC treatment, plasma-derived exosomes (SI-exo) were taken up by OFs, transferring miR-885-3p from the EVs into the OFs. Within OFs, miR-885-3p directly binds the 3’-UTR region of the AKT2 gene, achieving targeted suppression of AKT2 and inhibits the activation and phosphorylation levels of AKT. This, in turn, dampened activation of the pro-inflammatory AKT/NF-κB signaling pathway. As this axis was suppressed, glucocorticoid receptor (GR) expression GR-dependent glucocorticoid response element (GRE) activity increased, while inflammatory molecules including IL-1 and ICAM-1 were reduced. Together, these changes appear to enhance glucocorticoid responsiveness in the peripheral vascular layer, which may explain the improved clinical response to IVGC therapy. On this basis, miR-885-3p has been proposed as a potential biomarker for predicting treatment efficacy. However, the current evidence is largely derived from small, single-center studies and lacks robust validation in larger cohorts. For instance, the aforementioned study included only 17 patients, limiting statistical power and generalizability. In addition, the absence of key performance measures, such as sensitivity, specificity, and ROC curves, makes it difficult to judge diagnostic performance objectively. Future work should combine machine-learning based biomarker screening with ROC-based validation, and address potential confounders using multivariable regression (58–60). Reproducibility and clinical translation are also constrained by the lack of standardized protocols for exosome isolation and quantification (55). Therefore, despite encouraging mechanistic insights, substantial obstacles remain before such biomarkers can be adopted in practice. Large-scale, multicenter studies with standardized operating procedures and rigorous performance evaluation will be essential to move these candidates toward clinical use.

### 3.4 Proteomic profiling of tear-derived EVs

Tear derived exosomes from GO patients have increased concentrations and contain pro-inflammatory proteins such as caspase-3, complement C4A and apolipoprotein A-IV. These proteins indicate pathological changes of orbital tissues, which implies that tear EVs can be used as a noninvasive indicator for the disease monitoring. In addition, the increased concentrations of IL-18 and IL-1 in tear EVs suggest that they may be involved in local immune dysregulation and apoptosis. In Shi’s study, results demonstrated that patients with active/severe GO (defined as CAS ≥ 3, with cohort CAS values concentrated within a narrow range of approximately 3.6 ± 0.3) exhibited significantly upregulated IL-1 and IL-18 in tear exosomes, consistently validated in serum. This suggests that these inflammatory cytokine loads align with a “high-activity inflammatory phenotype”. However, this study did not perform direct statistical correlation between molecular levels and CAS on an individual case basis: most samples were pooled, and the cohort primarily comprised active-phase patients with high CAS, making it difficult to assess whether these factors vary along the CAS gradient. Therefore, to establish robust evidence linking ‘molecular load directly to clinical phenotype’ as a non-invasive biomarker, future studies should quantify EV-IL-1/IL-18 in individual patients without pooling samples. Spearman correlation and ROC analysis should then be performed to evaluate their discriminatory performance in distinguishing active/inactive phases (or different CAS grades) (56).

### 3.5 Functional effects on orbital fibroblasts

**Figure 2:**
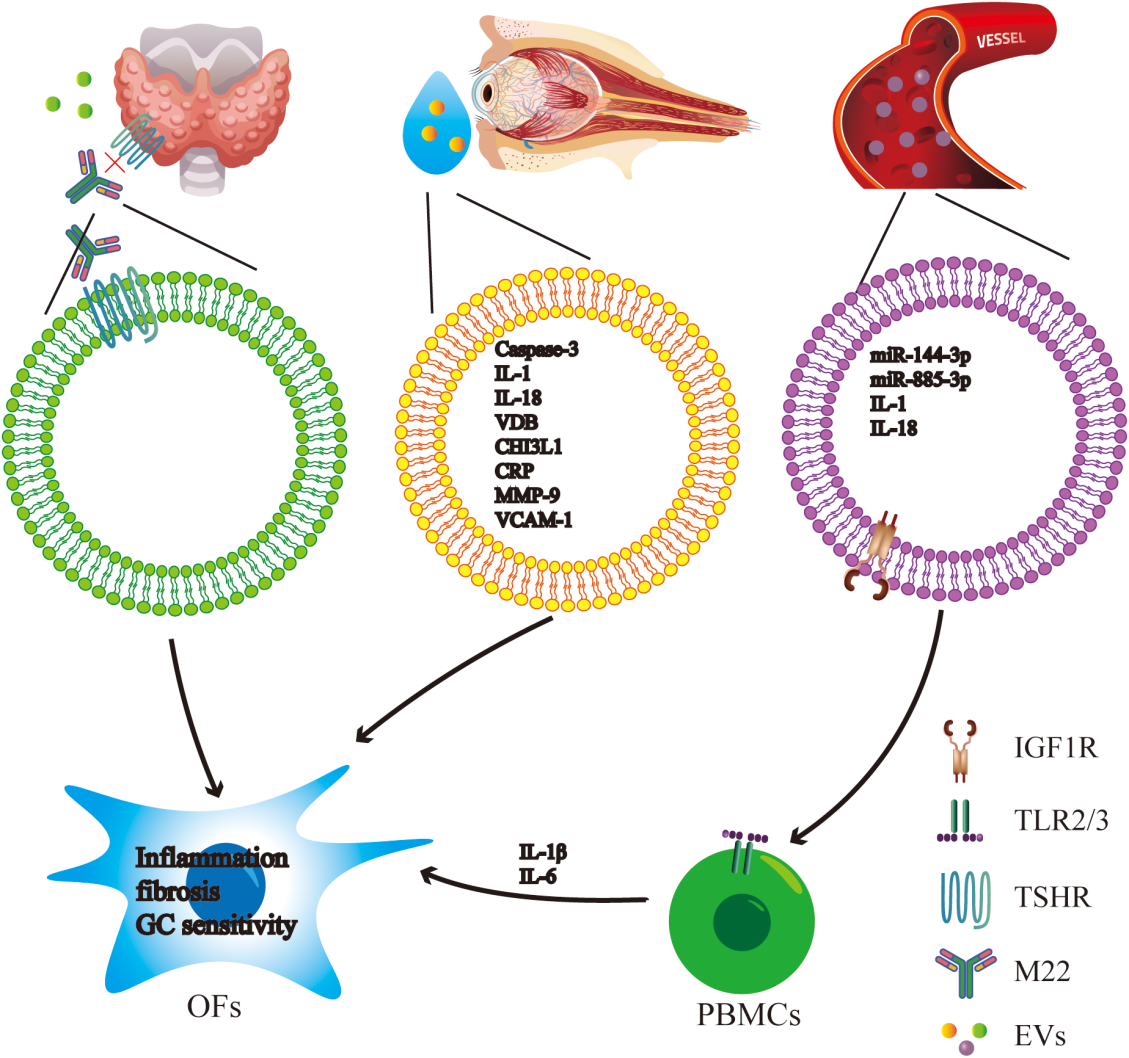
Mechanisms of EVs in GD and GO, and their potential as biomarkers. Schematic of EVs sources and downstream effects in GO and GD. Thyroid-derived EVs expressing TSHR may interact with stimulatory TSHR antibodies (e.g., M22), suggesting a potential “bait” mechanism to modulate antibody bioactivity. Tears-derived exosomes carry proteins involved in inflammation and tissue remodeling (e.g., caspase-3, IL-1, IL-18, VDB, CHI3L1, CRP, MMP-9, VCAM-1), underscoring their value as non-invasive biomarkers reflecting ocular surface and orbital inflammation. Circulating exosomes rich in inflammatory cargo and miRNAs (e.g., miR-144-3p, miR-885-3p, IL-1, IL-18) interact with PBMCs; Exosomal components (including IGF-1R and potential TLR ligands) are proposed to activate the TLR2/3 signaling pathway, promoting pro-inflammatory cytokine production (e.g., IL-1β, IL-6). Cytokines released by activated PBMCs, combined with the direct uptake of EVs by OFs, jointly participate in the pathogenesis of GO by enhancing inflammation, fibrosis, extracellular matrix remodeling, and altering OFs’ GC sensitivity.

Exosomes derived from the tears of patients suffering from GO cause OFs to produce a variety of inflammatory mediators. Proteomic analysis showed upregulation of vitamin D binding protein, chitinase 3-like protein 1, and matrix metalloproteinase-9, implicating EVs in tissue remodeling and immune cell recruitment (54). Together they point to many different ways that EVs are involved in GO. Their participation covers up the immune system activation, fibrotic remodeling as well as therapeutic responses modification (**Figure 2****).** The recognition of particular exosomal miRNAs and proteins as potential biomarkers gives a promising approach to improving the early detection, following the course of disease and tailoring individual treatment options for GO patients.

Although there is great progress, there are some significant problems that need to be paid attention to in the studies of EVs in GO. Firstly, EV populations are heterogeneous, while the inconsistency of isolation and purification procedures makes it difficult to achieve reproducibility of results and comparisons across studies. Lots of studies use bulk plasma or tear-derived EVs without strict subtype classification, which might make it hard to spot disease-linked EV subsets. Second, the functional validation of identified EV cargoes, e.g., miRNAs or proteins, often lacks mechanistic depth. Some miRNAs like miR-144-3p are regarded as biomarkers or potential therapeutics. But direct causal connections to clinical results stay tentative and want *in vivo* confirmation. Third, the vast majority of existing data are derived from small, single-center studies with a limited number of patients, and few studies have long-term follow-up. Lastly, only a handful of studies have looked at whether the EV profiles are different between active vs. inactive GO stages and between treatment responders and non-responders. To truly harness the potential of EVs for both diagnosis and therapy in GO, these methodological and clinical gaps must be addressed.

## 4 EVs in Graves’ disease

Beyond their well-established function in GO, EVs show considerable involvement in GD’s pathogenesis and development. Research has shown that it has a role in immune modulation, inflammatory signaling, and can be used as diagnosis and prognosis markers (**Table 2**).

**Table 2:** Summarization of studies investigating the role of EVs in the pathogenesis of GD.

Origin of EVs	Cargo of EVs	Biological function	EV isolation method	Group and sample size	Year	Reference
GD patients’ serum-derived Exos	IGF-1R and HSP60	Interacted with PBMCs via TLR2/3 and activated the MyD88/TRIF/NF-κB pathway, upregulating IL-6 and IL-1β, suggesting a pro-inflammatory mechanism.	Sequential Ultracentrifugation	GO patients (n=7), GD patients (n=26), HC (n=26)	2021	(53)
Refractory GD patients’ serum Exos	Unmentioned	Induced higher levels of IL-1β, TNF-α, and IL-6 in PBMCs.	Polymer Precipitation	Intractable GD (n=7), GD in remission (n=7), HC (n=7)	2016	(61)
Refractory GD patients’ serum Exos	let-7g-3p and miR-339-5p	Linking miRNA dysregulation to persistent inflammation via NF-κB and IL-17 pathways.	Polymer Precipitation	Intractable GD (n=7), GD in remission (n=7), HC (n=7)	2016	(61)
GD patients’ Exos	MAP1S, VAMP8, and CXCL7	Involved in immune regulation and cell death.	differential ultracentrifugation	GD patients (n=12), Hashimoto’s thyroiditis (n=10), HC (n=7)	2021	(62)
GD patients’ plasma-derived Exos	hsa_circRNA_000102	Correlated with TRAb levels, implicated in immune activation pathways, and may function through a circRNA-miRNA-mRNA regulatory axis.	differential ultracentrifugation	GD patients (n=20), HC (n=20)	2020	(63)
GD thyroid tissue-derived Exos	miR-375-3p and miR-7-5p	Distinguished GD from papillary thyroid cancer and showed promise as tissue-specific biomarkers for differential diagnosis.	differential ultracentrifugation	GD patients (n=5), papillary thyroid cancer (n=5), benign tissue (n=5)	2023	(64)
Thyroid cell-derived Exos	TSHR	Bound to the GD-specific monoclonal antibody M22 and inhibited M22-induced cAMP signaling, suggesting a “decoy effect” that may mitigate hyperstimulation in GD.	differential ultracentrifugation	Not applicable	2019	(65)

### 4.1 Immune activation and inflammatory signaling

Exosomes isolated from the serum of patients with refractory GD can activate peripheral immune cells. These exosomes induce PBMCs to release more pro-inflammatory cytokines, indicating heightened inflammatory responses. Analysis of microRNA within these exosomes shows increased amounts of specific miRNAs, such as let-7g-3p and miR-339-5p. These microRNAs can also activate the NF-kB, IL-17 signals necessary for the autoimmunity and inflammation of GD (61).

### 4.2 Molecular profiling of EVs cargo

Proteomic analysis by data-independent acquisition (DIA) method found 208 differentially expressed proteins in exosomes of GD patients, including MAP1S, VAMP8 and CXCL7, involved in immune activation and apoptosis are upregulated (62). CircRNA profiling showed that substantial enrichment of hsa_circRNA_000102 in the plasma exosomes of patients with GD. The circular RNA is positively correlated to thyroid-stimulating hormone receptor antibody levels, and may affect disease progression via a circRNA-miRNA-mRNA regulatory network (63).

### 4.3 Functional modulation and environmental influence

Thyroid follicular cells secrete exosomes with TSHR protein. These vesicles bind to the GD specific monoclonal antibody M22, acting as decoys and reducing receptor over stimulation and downstream cyclic adenosine monophosphate signaling (65). Environmental toxins (e.g., DDT) disrupt normal TSHR transport or exosome coating processes, potentially triggering autoimmune responses in susceptible individuals (66). The levels of plasma microvesicles and platelet-derived microparticles are increased in GD and Hashimoto’s thyroiditis patients. These vesicles contain inflammatory markers and keep a partial boost after treatment, suggesting their role in monitoring disease activity and immune system dysregulation (67, 68).

### 4.4 EVs as biomarkers and monitoring tools

The levels of plasma microvesicles and platelet-derived microparticles are elevated in patients with GD and HT and remain high before and after treatment. These particles carry inflammatory markers and may indicate disease activity or underlying immune dysregulation. In addition, some exosomal miRNAs and proteins show differential expression between GD and papillary thyroid carcinoma (PTC), including miR-375-3p and miR-7-5p, suggesting potential utility in distinguishing thyroid diseases (64). Overall, the current evidences support an ideal role for EVs in GD pathogenesis rather than viewing them as byproducts of immune activation. Beyond GD, EVs have also shown diagnostic potential across multiple disease settings, highlighting their promise as biomarkers, and therapeutic targets (69). Future works should confirm these signatures in larger cohorts and develop standardized protocols to support clinical translation.

## 5 EVs in other immune-mediated eye diseases

In addition to GD and GO, EVs have also been implicated in several other autoimmune eye diseases (70–72). These disorders are commonly characterized by immune imbalance and inflammation (73, 74). Importantly, the roles of EVs in these diseases overlap to some extent with those reported in GO. In autoimmune uveitis, for instance, EVs can act as pro-inflammatory carriers which trigger innate immune activation and propagate downstream inflammatory signaling. They can increase the inflammatory mediators such as IL-1β, IL-6, and TNF-α, thereby driving immune cell recruitment and intra-uveal inflammation. This aligns with the mechanism in active GO where EVs induce inflammatory responses in OFs and exacerbate disease progression (75). Second, similar to GO, EVs in Sjögren’s syndrome carry miRNA and protein cargo involved in adaptive immune remodeling, particularly through coupling with Th17/Treg imbalance and IL-17-associated inflammatory networks. Research suggests these are associated with disease activity, recurrence risk, or treatment sensitivity (76). Based on these shared mechanisms, the inflammatory cytokine profiles, complement/apoptosis-related proteins, and miRNA characteristics of exosomes derived from bodily fluids—such as plasma, tears, and saliva—serve as minimally invasive, cross-disease biomarkers for diagnosis, activity monitoring, and stratification. They also provide a theoretical foundation for exploring common intervention targets in GO/GD and other immune-mediated eye diseases by investigating “EVs-mediated immune communication”.

## 6 Conclusion and future perspectives

EVs play a key role in GD and GO pathogenesis, diagnosis and potential therapy. EVs drive immune cells to remodel tissues and deliver cell signals through transporting functional cargoes such as proteins and nucleic acids. Notably, EVs both represent the pathological state of their source cell types and impact the immune microenvironment of the thyroid and orbit. From a translational medicine perspective, specific miRNAs and proteins found in blood- and tear-derived exosomes are linked to disease activity and treatment outcomes. EVs exhibit potential in engineered targeted drug deliveries, and present a novel strategy for precision immunotherapy. Although research on EVs has advanced substantially, several challenges still need to be addressed. The major issue is the lack of standardized protocols for EVs isolation and characterization, which undermines reproducibility and makes it difficult to compare results across clinical studies given the intrinsic heterogeneity of EVs. Furthermore, the molecular basis underlying EV-mediated immune communication remains incompletely understood and warrants further investigations.

Before EVs can be used routinely in the clinic, several technical issues need to be solved. One practical challenge is the efficient isolation and reliable identification of EVs from limited tear volumes. Methods that minimize protein co-isolation will be particularly important. Another challenge is engineering EVs into orbit-targeted delivery vehicles. One possible route is to load the anti-fibrotic drugs, including teprotumumab or other IGF-1R inhibitors via electroporation or ultrasonication, then rigorously measure and improve the loading efficiency. As major knowledge gaps still remain, both *in vivo* and *in vitro* studies are needed to clarify how specific exosomal cargoes contribute to GO and GD. Future work should combine high-throughput omics, advanced imaging, and longitudinal multicenter cohorts to better define the diagnostic and therapeutic value of EVs. Progress will also depend on closer collaboration across endocrinology, ophthalmology, immunology, and bioengineering to translate these findings into personalized therapies for GO. Overall, EVs represent a promising platform for monitoring and treating GO and GD, but their clinical application will require sustained, in-depth interdisciplinary efforts.
